# Managing Oral Surgery in von Willebrand Disease: Lessons from a Challenging Case

**DOI:** 10.4317/jced.62620

**Published:** 2025-06-01

**Authors:** Giuliano Ascani, Paola Ranalli, Francesca Azzuni, Silvia Benfatto, Michele Romano, Erminia Di Nobile

**Affiliations:** 1 Department of Maxillofacial Surgery, Spirito Santo Hospital, Pescara, Italy; 2 University of Chieti – Pescara, Chieti, Italy; 3 Department of Hematology, Spirito Santo Hospital, Pescara, Italy; 4 University “Cattolica del Sacro Cuore”, Rome, Italy; 5 University “Federico II”, Naples, Italy; 6 Private Dental Practice, Chieti and Pescara, Italy

## Abstract

Von Willebrand disease is a rare inherited bleeding disorder characterized by deficient or defective von Willebrand factor, crucial for platelet adhesion and aggregation. This case report aims to highlight the challenges of diagnosing undiagnosed von Willebrand disease in dental practice and to describe a multidisciplinary management approach to prevent life-threatening complications during oral surgery. A 32-year-old male with no significant medical history underwent surgical extraction of an impacted third molar. The patient experienced severe intraoperative bleeding unresponsive to standard local hemostatic measures. Subsequent hematological evaluation confirmed Type I von Willebrand disease. For the extraction of the remaining impacted molars, a tailored protocol was implemented, including preoperative administration of von Willebrand factor-containing factor VIII concentrates, tranexamic acid therapy, local hemostatic agents, and close perioperative monitoring in collaboration with hematology specialists. The initial procedure resulted in excessive bleeding, leading to the diagnosis of von Willebrand disease. Following the implementation of the multidisciplinary protocol, the patient underwent multiple extractions without immediate or delayed bleeding complications. Hemoglobin levels remained stable postoperatively, and the patient reported satisfactory pain control. Early identification and tailored management of bleeding disorders are critical in oral surgery to prevent severe hemorrhagic complications. A multidisciplinary approach involving dentists, oral surgeons, and hematologists ensures safe and effective care for patients with von Willebrand disease, emphasizing the importance of comprehensive preoperative assessments and structured treatment protocols.

** Key words:**Bleeding disorders, Dental extraction, Hemostasis, Oral surgery, von Willebrand disease, Multidisciplinary management.

## Introduction

Von Willebrand disease (vWD) represents an inherited rare bleeding disorder, with an estimated prevalence of approximately 1% in the global population. Type I von Willebrand Disease, mostly transmitted as an autosomal dominant defect, accounts for the majority of all cases and it is due to a partial deficiency of von Willebrand Factor (vWF), a glycoprotein essential for platelet adhesion and aggregation, in contrast with Type II vWD (qualitative defect) and Type III vWD (almost complete absence of vWF); despite mucocutaneous bleeding is the most recurrent symptom associated with vWD, frequently patients with vWD, especially Type I vWD, are asymptomatic or have only mild symptoms; in previously undiagnosed cases, hemorrhagic complications occurring during surgical procedures, including oral surgery, often allow the diagnosis to be defined (1-3).

The challenges associated with managing patients with vWD in dentistry have been widely documented. A Cochrane review by van Galen *et al*. (1) emphasized the efficacy of antifibrinolytic agents, such as Tranexamic Acid (TXA) or Epsilon Aminocaproic Acid (EACA), in reducing postoperative bleeding in patients with hemophilia undergoing dental extractions, suggesting that similar strategies could benefit vWD patients. Additionally, Watterson *et al*. (2) underscored the importance of tailored protocols for the dental management of patients with bleeding disorders, reinforcing the need for further research to develop standardized, evidence-based guidelines.

Furthermore, Rodeghiero *et al*. (3) explored the diagnostic challenges of vWD and highlighted the variability in clinical manifestations, which often results in delayed or missed diagnoses. This variability underscores the importance of accurate preoperative assessment and medical history review in patients undergoing surgical procedures, including dental extractions.

This case report illustrates the significance of recognizing and managing undiagnosed vWD in dental practice to prevent life-threatening complications. It also highlights the need for a multidisciplinary collaboration between dentists, oral surgeons, and hematologists to ensure optimal patient outcomes.

## Case Report

**Figure 1:**
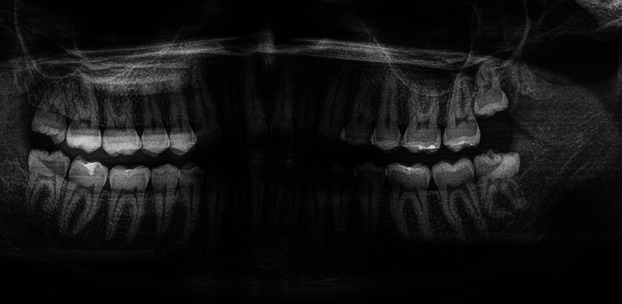
Preoperative orthopantomogram.

A 32-year-old male patient presented for routine dental care, reporting no significant medical history, medication use, or prior surgical interventions. The patient exhibited good periodontal health aside from impacted third molars, necessitating surgical extractions (teeth 1.8, 2.8, 3.8, and 4.8) (Fig. 1).

The extraction of tooth 3.8 was performed in an outpatient setting. During the procedure, the patient experienced prolonged and excessive bleeding, unresponsive to conventional local hemostatic measures such as gauze compression and hemostatic agents (Adsorbable Haemostatic Geletin Sponge). Bleeding was eventually controlled by applying regenerated fibrillar oxidized cellulose to the surgical wound and by compressing it with gauze saturated with tranexamic acid for thirty minutes. After a three-hour observation period, the patient was discharged with detailed postoperative instructions, and outpatient clinical evaluations were performed every two days for one week. No complications occurred during the observation period, and the sutures were removed on the tenth postoperative day. Given the severity of intraoperative hemorrhage, the patient was referred to the designated secondary-level referral hospital, Spirito Santo Hospital of Pescara, where he was managed by the Maxillofacial Surgery and Hematology Departments according to the standardized Diagnostic-Therapeutic Care Pathway (PDTA). The PDTA (Percorso Diagnostico Terapeutico Assistenziale) is the internal multidisciplinary protocol used at the hospital for managing patients with hemorrhagic disorders, from diagnosis to treatment and follow-up.

Family history revealed an increased bleeding tendency in patient’s mother and sister.

Despite first level hemostatic tests did not show any abnormalities (PT, PTT, fibrinogen) in our patient, further tests were performed by our local hemostasis laboratory, including enzyme-linked immunosorbent assays measuring vWF antigen (vWF:Ag: 29%) and activity, this latter measured with RICOF assay (Ristocetin Cofactor Activity: 28%)), resulting suggestive for a diagnosis vWD. Type I vWD diagnosis was confirmed according to RICOF/vWF:ag ratio (0.96) and vWF multimer testing, showing vWF all size multimers present, despite in reduced concentrations.

**Figure 2:**
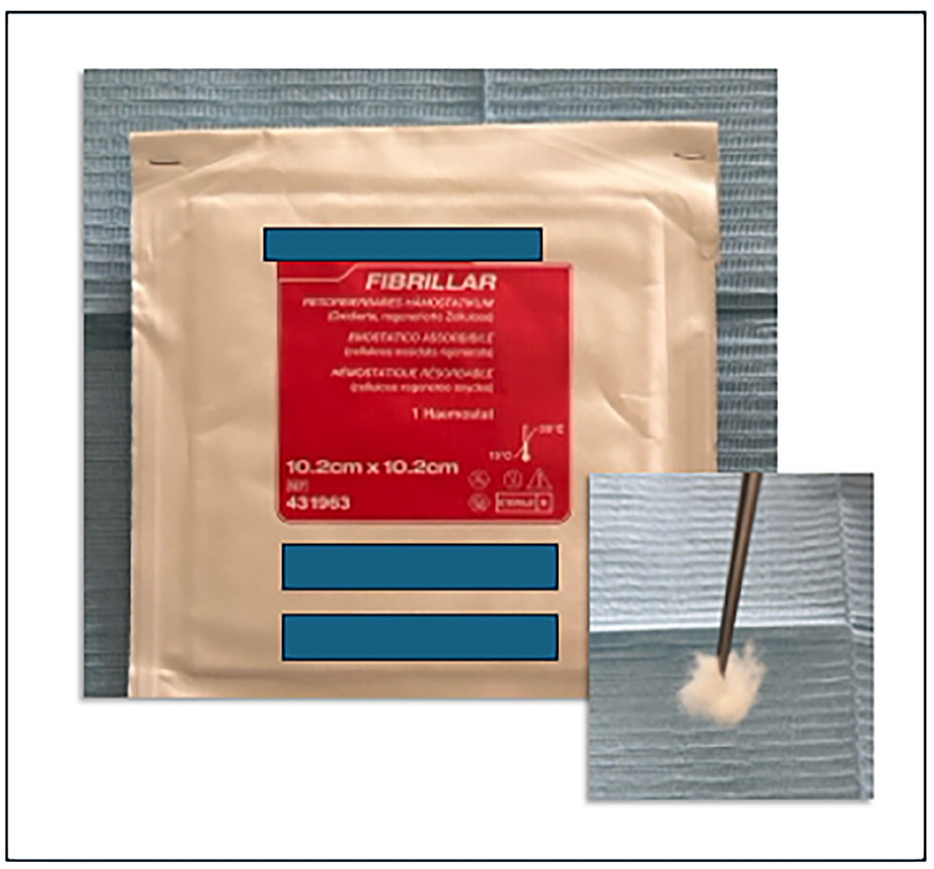
Local hemostatic agent.

In subsequent months, extractions of the remaining impacted molars (teeth 1.8, 2.8, and 4.8) were performed following a carefully structured protocol, including:

o Tranexamic acid (Tranex) 25 mg/kg in oral Tablets every 8 hours (single dose: 1500 mg), starting the evening before the procedure.

o Preoperative intravenous administration of plasmaderived FVIII concentrates enriched with von Willebrand Factor (Haemate P 50 UI/weight kilograms) 30 minutes before starting procedure.

o Use of local hemostatic agents, such as fibrin glue and oxidized regenerated cellulose (Fig. 2)

o Use of liquid Tranexamic Acid vials for local application (compression with gauze impregnated with Tranexamic Acid), if needed

o Oral antibiotic therapy as standard surgical indications.

o Close postoperative in-hospital monitoring (4 hours lasting), before discharge on the same day.

o In case of bleeding, the patient was invited to refer to Our Emergency Department.

o The day after the procedure: blood count and plasmaderived FVIII concentrates + vWF Factor (Haemate P 50 UI/weight kilograms, 3.000 UI overall) was administered for further two consecutive days at Hemophilia Centre

o Follow-up visit at the Maxillofacial Surgery Department for post-surgical control.

For the Maxillofacial Surgery Department the protocol for management of patients with bleeding disorders expected that:

• The procedure date (avoiding Fridays if possible) was notified and shared with the Hemophilia Center in order to better manage bleeding complications eventually occurring after surgery and to plan subsequent infusions of factor concentrate.

• Provide prescriptions for factor concentrate, ensuring the patient has an adequate supply (at least 9,000 IU overall).

**Figure 3:**
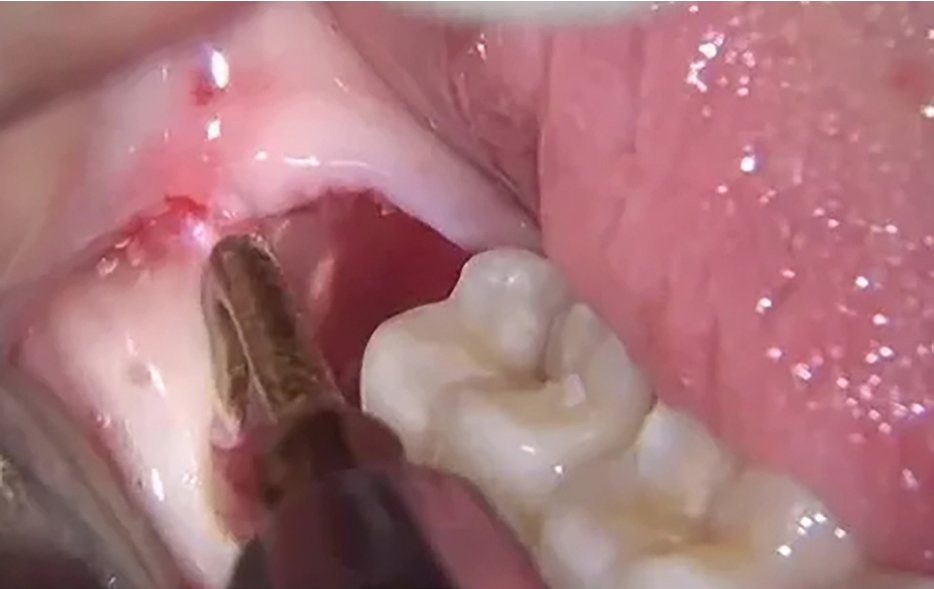
Intraoperative photo (surgical extraction of 4.8) showing good hemostasis.

These measures resulted in successful multiple extractions without immediate (Fig. 3) and/or delayed bleeding complications. Pre-procedural hemoglobin levels were unmodified after procedure (hb 13.7 → 13.4 g /dL). Control pain was satisfying, according to patient’s reported experience.

## Discussion

This case underscores the importance of early identification and tailored management of bleeding disorders in oral surgery. The lack of an early vWD diagnosis in this patients led to severe intraoperative hemorrhage, demonstrating the need for heightened clinical suspicion when excessive bleeding occurs unexpectedly (4,5).

Standard coagulation tests such as PT, PTT, and fibrinogen levels provide valuable information about the coagulation cascade, yet they have noTable limitations in diagnosing von Willebrand Disease (VWD). For instance, the prothrombin time (PT) typically remains within the normal range in VWD patients, as this test primarily reflects the extrinsic and common coagulation pathways rather than the function of von Willebrand factor. Similarly, the partial thromboplastin time (PTT) may be only mildly prolonged or even normal, depending on the severity and subtype of VWD, and does not specifically assess the qualitative aspects of von Willebrand factor that are crucial for platelet adhesion and aggregation. Fibrinogen levels, on the other hand, are not affected by VWD, making them an unreliable marker for the disease. Consequently, while these tests can rule out broader coagulopathies, they lack the sensitivity and specificity needed to accurately diagnose VWD. Specialized assays—such as von Willebrand factor antigen, ristocetin cofactor activity, and multimer analysis—are essential to capture the nuances of VWD pathology and to provide a comprehensive evaluation of both quantitative and qualitative defects in von Willebrand factor (5,6).

Malmquist (7) highlighted the role of structured preoperative preparation, including hemostatic support, in minimizing bleeding risks in surgical patients with vWD. Our case reinforces these findings and suggests that implementing standardized screening protocols for bleeding disorders in at-risk patients may improve patient safety and surgical outcomes.

Key clinical considerations include:

• Preoperative Assessment: Dentists and oral surgeons should obtain detailed bleeding histories, including familial tendencies, easy bruising, and prolonged bleeding from minor injuries.

• Surgical Planning: Patients with confirmed or suspected vWD should undergo preoperative laboratory testing and hematology consultation to guide individualized treatment approaches.

• Postoperative Monitoring: Close follow-up is essential to detect delayed bleeding events, which are common in vWD patients due to impaired clot stability.

A case report by Baghaie *et al*. (8) described a similar situation in which a patient with undiagnosed vWD developed significant postoperative bleeding following third molar extraction. This further highlights the necessity for dental professionals to remain vigilant for undiagnosed coagulopathies and to implement immediate hemostatic measures when excessive bleeding occurs.

## Conclusions

Early diagnosis is crucial for the effective management of patients with bleeding disorders in dentistry. A comprehensive medical history that includes detailed inquiries into previous bleeding episodes is essential for identifying at-risk patients before dental procedures. In our case, the early recognition of abnormal bleeding patterns led to a definitive diagnosis of von Willebrand disease, underscoring the critical role of vigilant preoperative assessment.

The effectiveness of the structured protocol—featuring the preoperative administration of Haemate P, tranexamic acid therapy, and rigorous perioperative monitoring—was clearly demonstrated by the successful management of subsequent extractions without hemorrhagic complications. This approach not only ensured patient safety but also optimized resource utilization by preventing emergency interventions.

A comprehensive multidisciplinary approach integrating dentists, maxillofacial surgeons, and hematologists is indispensable for ensuring safe and effective care. Furthermore, our findings highlight the urgent need to establish standardized protocols for managing patients with bleeding disorders in oral surgery. Such protocols should incorporate early diagnostic strategies, tailored hemostatic measures, and close interdisciplinary collaboration.

Finally, continuing education and training programs focused on the recognition and management of coagulopathies are essential for improving clinical outcomes and reducing surgical risks. Concentrating oral surgery expertise in specialized centers closely connected to local Centers for Congenital Bleeding Disorders is advisable to provide the best possible care to patients suffering from these rare disorders.

## Data Availability

The datasets used and/or analyzed during the current study are available from the corresponding author.
